# Investigation of the relationship between the mandibular third molar teeth and the inferior alveolar nerve using posteroanterior radiographs: a pilot study

**DOI:** 10.1186/s12903-024-04123-x

**Published:** 2024-03-22

**Authors:** Burak Kerem Apaydın, Derya Icoz, Ezgi Uzun, Kaan Orhan

**Affiliations:** 1 Department of Oral and Maxillofacial Radiology, Faculty of Dentistry, Pamukkale University, Denizli, 20160 Turkey; 2 Department of Oral and Maxillofacial Radiology, Faculty of Dentistry, Selcuk University, Konya, 42100 Turkey; 3 Department of Oral and Maxillofacial Radiology, Faculty of Dentistry, Ankara University, Ankara, 06500 Turkey; 4 Medical Design Application and Research Center (MEDITAM), Ankara University, Ankara, 06500 Turkey; 5 Department of Oral Diagnostics, Faculty of Dentistry, Semmelweis University, Budapest, Hungary

**Keywords:** Mandibular third molar, Third molar complication, Inferior alveolar nerve, Radiographic examination, Panoramic radiography, Posteoanterior radiography, Cone beam computed tomography

## Abstract

**Background:**

The most severe complication that can occur after mandibular third molar (MM3) surgery is inferior alveolar nerve (IAN) damage. It is crucial to have a comprehensive radiographic evaluation to reduce the possibility of nerve damage. The objective of this study is to assess the diagnostic accuracy of panoramic radiographs (PR) and posteroanterior (PA) radiographs in identifying the association between impacted MM3 roots and IAN.

**Methods:**

This study included individuals who had PR, PA radiographs, and cone beam computed tomography (CBCT) and who had at least one impacted MM3. A total of 141 impacted MM3s were evaluated on CBCT images, and the findings were considered gold standard. The relationship between impacted MM3 roots and IAN was also evaluated on PR and PA radiographies. The data was analyzed using the McNemar and Chi-squared tests. The sensitivity, specificity, positive predictive value (PPV), negative predictive value (NPV), and diagnostic accuracy of PR and PA radiographies were determined.

**Results:**

Considering CBCT the gold standard, the relationship between MM3 roots and IAN was found to be statistically significant between PR and CBCT (*p* = 0.00). However, there was no statistically significant relationship between PA radiography and CBCT (0.227). The study revealed that the most prevalent limitation of the PR in assessing the relationship between MM3 roots and IAN was the identification of false-positive relationship.

**Conclusions:**

PA radiography may be a good alternative in developing countries to find out if there is a contact between MM3 roots and IAN because it is easier to get to, cheaper, and uses less radiation.

## Background

An impacted tooth refers to a tooth that is unable to emerge into the dental arch within the anticipated timeframe due to a physical obstruction, insufficient space, or improper positioning [1]. Mandibular third molars (MM3s) have the highest likelihood of becoming impacted [2]. Numerous reasons have been elucidated in the literature regarding the impaction of the lower third molars. The most common causes of impaction of the lower third molar include abnormal positioning of the tooth germ, insufficient space in the dental arch, ankylosis of the deciduous or permanent tooth, bone that does not resorb due to local or systemic reasons, bone obstruction along the eruption path, or obstruction by adjacent teeth, and various syndromes [3]. Various etiological factors may contribute to the impaction of third molars, but craniofacial development is certainly one of the most significant factors. It has been reported that there is a correlation between the impaction classification types of lower third molars and ramus height/gonial angle. A lower gonial angle was found to be significantly associated with a higher prevalence of impaction of the lower third molars. A decreased gonial angle is associated with a reduced retromolar space, thereby supporting the impaction of the lower third molars and their closer proximity to IAN during development [4, 5]. Impacted MM3s can lead to a variety of problems, including caries, pericoronitis, resorption, bone loss in the distal surface of the second molar, cystic or neoplastic conditions, and myofascial and neurogenic pains [1, 6] Furthermore, impacted MM3s may cause temporomandibular joint disorders, dental problems like crowding, weaken the mandibular angle, and potentially cause fractures [2].

Surgical removal of impacted MM3s is a frequently performed operation in oral and maxillofacial surgery for prophylactic, therapeutic, and orthodontic reasons [1, 7]. The extraction of impacted MM3s can lead to various complications, such as swelling, pain, infection, excessive bleeding, and reduced mouth opening [8–10]. However, one of the most severe postoperative complications is the injury of the inferior alveolar nerve (IAN), which results in reduced sensation in the lower lip and mandible. Postoperative sensory impairment might exhibit either permanent or temporary characteristics [1, 11]. If individuals with IAN injuries do not show spontaneous healing within a period of 6 months, the IAN damage is deemed to be permanent [12]. According to various studies, the incidence of cases with permanent IAN damage has been reported as less than 1% [13, 14]. The occurrence of temporary nerve injuries is often estimated to range from 0.4 to 8% [13, 14]. However, in cases where there is close proximity between the MM3 roots and IAN, the incidence can be as high as 30% [13]. IAN damage may affect patients’ quality of life by causing mental and social problems [13, 15]. It has also been reported that this is the most common cause of complaints against oral and maxillofacial surgeons in forensic courts, increasing the public’s belief that surgical negligence occurs during surgery [13].

The incidence of IAN damage is influenced by several factors, including the surgeon’s experience, the gender and age of the patient, the type of anesthesia, and the anatomical relationship between the mandibular canal and MM3 [12]. In contrast, Ghaeminia et al [16] reported that there is no significant relationship between IAN injury and the experience of the surgeon, age, or gender of the patient. It has been suggested that the close proximity of the MM3 roots to the IAN and their positional relationship are the most important risk factors. IAN injury can occur during the surgical removal of MM3s due to many factors, such as indirect compression, insufficient bone cortex around the IAN, or direct trauma. When the MM3s anatomically contact the IAN, the risk of IAN damage increases after tooth extraction [17]. To limit this risk, it is important to determine the location of the IAN relative to the MM3 roots before the surgery using a radiographic examination [11].

For an optimal radiographic assessment of impacted MM3s, it is important to assess dental features such as root development, morphology, and number of roots. Additionally, the relationship between the impacted tooth and the surrounding bone, neighboring teeth, and anatomical structures should be assessed [18]. There is no specific protocol for the radiographic evaluation of MM3s [19]. Panoramic radiographs (PRs), which are the most commonly used in radiographic examination, are the first choice of dentists in determining the relationship between MM3 roots and the IAN due to their short scanning time, low radiation dose, and easy accessibility [17]. Furthermore, PR is also employed to assess the root morphology, degree of impaction, and angulation of the MM3s [20]. Nevertheless, PR has drawbacks, including poor image resolution, anatomical noise, overlapping of structures, geometric distortion, and the occurrence of phantom images [1, 17]. Establishing the connection between MM3 roots and IAN on PR might be challenging owing to the overlapping of the roots on the IAN, particularly when tooth roots are positioned on the buccal or lingual side of the IAN [17]. Hence, the exact anatomical relationship between MM3s and IAN cannot be determined using PRs [20]. Rood and Shehab [21] proposed the utilization of seven radiographic indicators that demonstrate the proximity between MM3 and IAN in PR. If a relationship between MM3 roots and IAN in PR is suspected based on these signs, it may be necessary to evaluate the relationship with cone beam computed tomography (CBCT). CBCT, which is one of the advanced imaging methods, provides a three-dimensional representation of the relationship between impacted MM3s and IAN, which contributes to simplifying preoperative planning and minimizing the likelihood of IAN damage [17]. However, CBCT is not commonly employed as a routine radiographic examination because of its limited accessibility, high cost, and radiation exposure [15, 17].

The posteroanterior (PA) radiograph is the second most commonly utilized skull radiograph in dentistry [22]. PA radiographs are necessary for assessing the transverse dimensions of the dentoalveolar structures and craniofacial skeleton [23]. For all that, it has been reported that PA radiographs can be utilized to determine the relationship between impacted MM3 roots and IAN [18].

The utilization of the CBCT technique is widely regarded as the most reliable method for assessing the relationship between the roots of MM3 and IAN [24]. The aim of this study is to evaluate the reliability of PR and PA radiographs in detecting the relationship between impacted MM3 roots and IAN. The null hypothesis of this study was that ‘there is no statistically significant difference between PR and PA radiographs in detecting the relationship between impacted MM3 roots and IAN’.

## Methods

This study was carried out in accordance with the principles outlined in the Declaration of Helsinki and received ethical approval from the Pamukkale University Non-Interventional Clinical Research Ethics Committee (E-60116787-020-374406). The sample size was calculated for the effect size (d, effect size = 0.85), type I error (α = 0.05), and 85% power values, the sample size was determined to be at least 80 for the study group.

Individuals who had all PR, PA radiography, and CBCT images obtained in the same week in the Pamukkale University Faculty of Dentistry archive and had at least one impacted MM3 were included in the present study. Images displaying incomplete root development and bone pathology in the evaluated area were omitted from the study. For the study on the CBCT images of 83 patients (25 males, accounting for 30.12%, and 58 females, accounting for 69.88%), a total of 141 impacted MM3s were evaluated, and CBCT was considered the gold standard. The PR and PA radiographs were obtained with the same device (Instrumentarium OP 200D, Tuusula, Finland), and CBCT images were obtained with Newtom 5G XL (Cefla, Imola, Italy) following the instructions provided by the manufacturer.

**Fig. 1:**
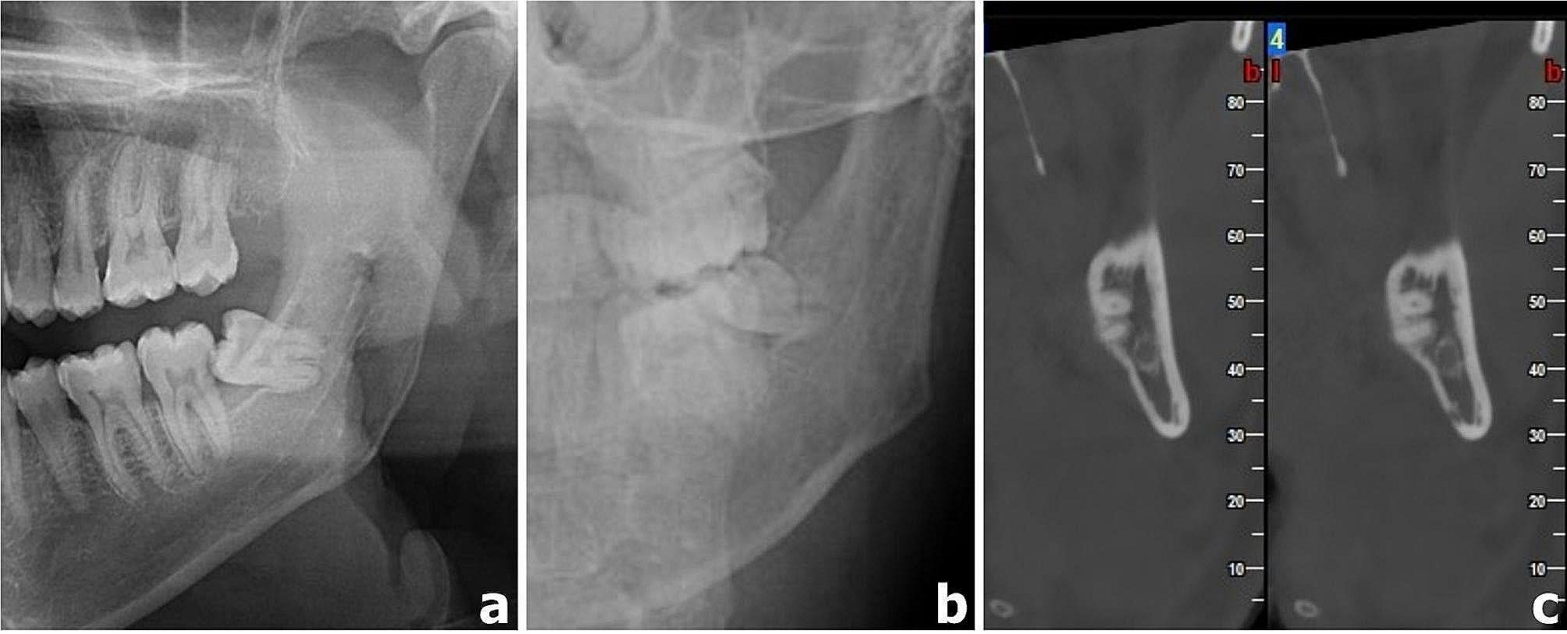
Cropped PR showing the roots of MM3 in relation to the IAN **(a)**, cropped PA radiograph **(b)**, and cross sectional CBCT images **(c)** of the same patient showing the absence of a relationship between MM3 roots and IAN

**Fig. 2:**
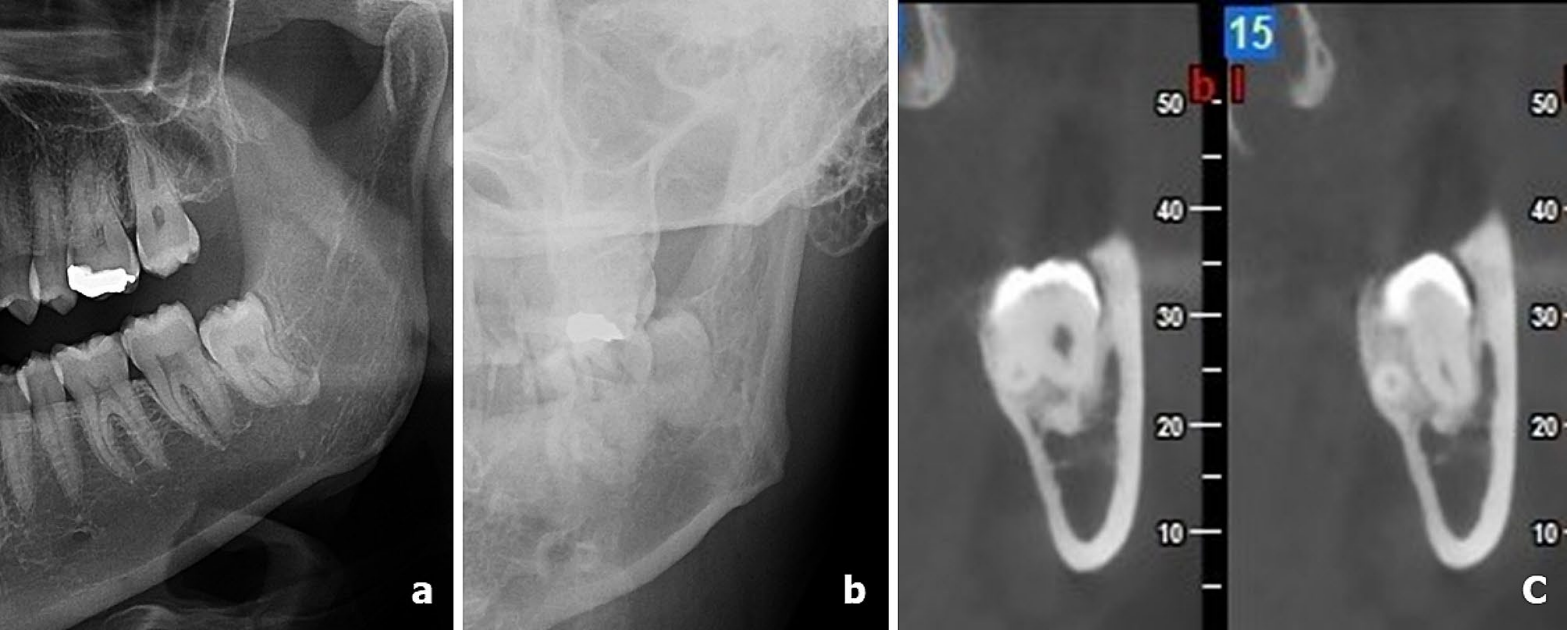
Cropped PR **(a)**, cropped PA radiography **(b)**, and cross sectional CBCT images **(c)** showing the presence of a relationship between MM3 roots and IAN

The angulations of the impacted MM3s, the locations of the impacted MM3 roots according to the IAN, and the presence of contact between the IAN and the impacted MM3s were evaluated on CBCT images. Two observers, one with 2 years of experience and the other with 13 years of experience in dentomaxillofacial radiography, assessed these factors (Figs. 1 and 2). The angulations of the impacted MM3s were evaluated according to the classification system of Winter as vertical, mesioangular, horizontal, distoangular, buccolingual, and others on all imaging techniques included in the study [25].

The location of the MM3 roots was classified as at the lingual, interradicular, buccal, and inferior of the IAN on the CBCT images [26]. The presence of the relationship between impacted MM3 roots and IAN was also evaluated on PR and PA radiographies independently by the same two observers under dim lightning conditions on a medical monitor (Barco MDNC-2221monitor, resolution 1600 × 1200, size 432 × 324, Barco, Kortrijk, Blegium), and inter-observer agreement was evaluated.

### Statistical analysis

The data was analyzed by using SPSS version 22.0 (SPSS Inc., Chicago, IL, USA). Inter-observer agreement was analyzed with the Cohen’s kappa test. Kappa values less than or equal to 0 were classified as indicating no agreement. Values ranging from 0.01 to 0.20 were considered to indicate no to minor agreement, while values between 0.21 and 0.40 were classified as fair agreement. Kappa values ranging from 0.41 to 0.60 were considered to indicate moderate agreement, while values between 0.61 and 0.80 were classified as significant agreement. Finally, values between 0.81 and 1.00 were considered to indicate practically perfect agreement [27]. Descriptive statistics were carried out, and results on categorical measurements were given as numbers and percentages. The data was analyzed using McNemar and Chi-squared tests. *p* ≤ 0.05 values were considered statistically significant. Sensitivity, specificity, positive predictive value (PPV), negative predictive value (NPV), and diagnostic accuracy of PR and PA radiographies were determined.

Sensitivity: True positive (TP)/(False negative(FN) + TP)

Specificity: True negative (TN)/(False positive (FP) + TN)

PPV: TP/(TP + FP)

NPV: TN/(FN + TN)

Diagnostic accuracy: (TP + TN)/(TP + FP + TN + FN)

## Results

The present study sample consisted of 141 impacted third molars from 83 individuals, whose ages ranged from 18 to 42, with a mean age of 25.95 ± 7.9 years. Out of the total number of teeth, 76 (53.9%) were found in the left mandible, while 65 (46.1%) were found in the right mandible. The evaluations on CBCT images were performed with consensus by two observers with 2 and 13 years of experience in dentomaxillofacial radiology. PR and PA radiography were evaluated independently by two observers, and kappa values were 0,862 and 0,815, respectively. The different assessments were re-evaluated by two observers, and the final decision was made. According to the results of the kappa analysis, the interobserver agreement was found to be almost perfect.

Table 1 shows the distribution of angulation types of MM3s on PR, PA radiography, and CBCT images. When the teeth were evaluated according to angulation, the highest canal relationship was seen in the buccolingual position, followed by the horizontal position. According to the location of roots relative to IAN, the presence of a relationship between IAN and impacted MM3 roots were most common in the lingual and interradicular positions, respectively. The difference between PR and PA radiographs was statistically significant in terms of the angulation of impacted MM3s (*p* = 0.00). The distribution of the presence of a relationship between MM3 roots and IAN according to angulations and the locations of the MM3 roots relative to IAN is seen in Tables 2 and 3. When the predictability of the relationship between MM3 roots and IAN was evaluated for PR and PA radiography according to the CBCT as gold standard, while the difference between PR and CBCT assessments was found to be statistically significant (*p* = 0.00), there was no significant difference between PA radiography and CBCT (*p* = 0.227). According to the results of our study, false positive relationship diagnosis was the most common handicap of the PR in evaluating contact between MM3 roots and IAN (Table 4).

**Table 1:** The distribution of the angulation types of mandibular impacted third molars according to imaging methods

	PR				PA radiography				
**CBCT**	Vertical	Mesio- angular	Horizontal	Bukko- lingual	Vertical	Mesio- angular	Horizontal	Bukko- lingual	Total
**Vertical**	83 (100%)	0 (0%)	0 (0%)	0 (0%)	81 (97.6%)	2 (2.4%)	0 (0%)	0 (0%)	83 (58.9%)
**Mesio-angular**	11 (28.9%)	27 (71.1%)	0 (0%)	0 (0%)	6 (15.8%)	30 (78.9%)	2 (5.3%)	0 (0%)	38 (27%)
**Horizontal**	0 (0%)	2 (12.5%)	14 (87.5%)	0 (0%)	0 (0%)	1 (6.3%)	15 (93.8%)	0 (0%)	16 (11.3%)
**Bukko-lingual**	1 (25%)	2 (50%)	0 (0%)	1 (25%)	0 (0%)	0 (0%)	0 (0%)	4 (100%)	4 (2.8%)
**Total**	95 (67.4%)	31 (22%)	14 (9.9%)	1 (0.7%)	87 (61.7%)	33 (23.4%)	17 (12.1%)	4 (2.8%)	**141** (100%)
**p**	0.000^*^				0.000^*^				

**Table 2:** The distribution of the presence of a relationship between IAN and impacted MM3 roots according to the angulations of the third molars

	PR		PA radiography		CBCT		Total
	Present	Absent	Present	Absent	Present	Absent	
**Vertical**	70 (84.3%)	13 (15.7%)	41 (49.4%)	42 (50.6%)	36 (43.4%)	47 (56.6%)	83 (58.9%)
**Mesioangular**	35 (92.1%)	3 (7.9%)	20 (52.6%)	18 (47.4%)	20 (52.6%)	18 (47.4%)	38 (27%)
**Horizontal**	15 (93.8%)	1 (6.3%)	13 (81.2%)	3 (18.8%)	13 (81.2%)	3 (18.8%)	16 (11.3%)
**Bukkolingual**	3 (75%)	1 (25%)	4 (100%)	0 (0%)	4 (100%)	0 (0%)	4 (2.8%)
**Total**	123 (87.2%)	18 (12.8%)	78 (55.3%)	63 (44.7%)	73 (51.8%)	68 (48.2%)	141 (100%)

**Table 3:** The distribution of the presence of a relationship between IAN and impacted MM3 roots according to the location of roots relative to IAN on CBCT images

	PR		PA radiography		CBCT		Total
	Present	Absent	Present	Absent	Present	Absent	
**Lingual**	29 (100%)	0 (0%)	28 (96.6%)	1 (3.4%)	29 (100%)	0 (0%)	29 (20.6%)
**Buccal**	44 (97.8%)	1 (2.2%)	21 (46.7%)	24 (53.3%)	19 (42.2%)	26 (57.8%)	45 (31.9%)
**Interradicular**	5 (83.3%)	1 (16.7%)	4 (66.7%)	2 (33.3%)	5 (83.3%)	1 (16.7%)	6 (4.3%)
**Inferior**	45 (73.8%)	16 (26.2%)	25 (41%)	36 (59%)	20 (%32.8)	41 (%67.2)	61 (43.3%)
**Total**	123 (87.2%)	18 (12.8%)	78 (55.3%)	63 (44.7%)	73 (%51.8)	68 (%48.2)	141 (100%)

**Table 4:** Positive and negative predictability results of the relationship between impacted MM3 and IAN according to PR and PA radiographies were diagnosed by CBCT as the gold standard

	PR		PA radiography		Total
**CBCT**	Positive	Negative	Positive	Negative	
**Positive**	70	3	70	3	73
**Negative**	53	15	8	60	68
**Total**	123	18	78	63	141
**p**	**0.000** ^*^		0.227		

The diagnostic accuracy of PR and PA radiography was 0.6 and 0.92, respectively, which indicates that the the presence of the relationship between MM3 roots and IAN is predictable at 60% in PR and 92% in PA radiography. When the diagnostic performance of the two imaging modalities was evaluated, the most significant difference was seen to be specificity at 0.22 and 0.88, respectively, for PR and PA radiography. PPV (0.56) and NPV (0.83) were also lower in PR compared to PA radiography. The sensitivity of both PR and PA radiographies was 0.96, which states that predictability is high for both imaging methods when there is a contact. (Table 5).

**Table 5:** Sensitivity, specificity, PPV and NPV values for PR and PA radiography determined according to CBCT as the gold standard

	Sensitivity	Specificity	PPV	NPV	Diagnostic accuracy
**PR**	0.96	0.22	0.57	0.83	0.6
**PA Radiography**	0.96	0.88	0.9	0.95	0.92

## Discussion

Before deciding to remove the MM3s, taking a radiographic image allows for the evaluation of the whole tooth, the bone around it, and its relationship with adjacent anatomical tissues. This aids the oral and maxillofacial surgeon in selecting the most suitable surgical approach [7, 19]. Oral and maxillofacial surgeons must inform the patient about the likelihood of IAN injury that may occur during the extraction of MM3s [19].

In this study, the Winter [25] classification was used to evaluate the angulation of impacted MM3s. Vertical position was the most common type, followed by mesioangular, horizontal, and buccolingual, respectively. This result is in disagreement with some other studies, which found the mesioangular position most common [14, 20, 28–30]. However, similar to this study, there are also studies showing that the vertical position is the most common, followed by the mesioangular position [30, 31].

The proximity of the MM3 roots to the mandibular canal may cause injury to IAN during the extraction of MM3s [32]. Paresthesia, albeit transient, is the primary source of discomfort and concern for patients [33]. Hence, a comprehensive radiographic assessment of this anatomical relationship is essential to apprise the patient of the potential hazards and formulate suitable surgical strategies [19, 20].

Currently, there is no definitive protocol for pre-extraction radiographic examination of MM3s, and therefore, there are no strict guidelines for dentists to follow. However, dentists should not perform surgery on MM3s without adequate radiographic evaluation. Without sufficient imaging procedures, a correct diagnosis cannot be made and may adversely affect the decision on appropriate treatment. Pathological findings may go unnoticed. In order to ensure thorough and well-informed consent, it is necessary to have radiological findings and clinical information that substantiate the pathology and associated risks. Hence, despite the lack of definitive evidence on the reduction of morbidity or complication rate, it is imperative to thoroughly investigate the region by radiographic imaging prior to surgical intervention [18].

Despite the disadvantages of magnification, distortion, and overlapping, PRs are the first choice of dentists to assess the surrounding anatomy of MM3s and the IAN due to their short scanning times, low radiation dosage, and easy accessibility [12, 17]. However, it is indeterminable whether the IAN course is positioned buccally or lingually of the MM3 roots or between the roots with PR [34]. Tantanapornkul et al. [35] found that the sensitivity and specificity of PR were 70% and 63%, respectively, and concluded that CBCT is significantly superior to PR for predicting the relationship between IAN and MM3s. In a study conducted by Sedaghatfar et al. [14] the researchers assessed the sensitivity and specificity of several predictors on PR. The sensitivity rates ranged from 17 to 71%, while the specificity rates ranged from 66 to 91%. However, in the case of the study in question, it was not specified which relationship type should be preferred in estimating the existence of exposure. The study conducted by Bell et al. [36] found that the sensitivity and specificity values for assessing the relationship between MM3 roots and IAN on PR are %66 and %74, respectively. The study found that the sensitivity of PR in predicting the contact between MM3 roots and IAN was 96%, whereas the specificity was 22%. In research conducted by Gomes et al. [37], it was shown that 61% of the cases had symptoms suggesting a connection between IAN and MM3s in PR. However, only 3.5% of the patients experienced paresthesia, and PR was not able to predict neurosensory problems.

On the other hand, several authors have reported that radiographic signs observed in PR, which serve as indications of the close anatomical relationship between MM3 roots and IAN, can be valuable in predicting IAN sensory impairment before surgery [15, 21, 26, 32]. The known classifications made on 2D radiographs by authors such as Winter, Pell&Gregory, and Rood&Shehab are still widely used for risk assessment in the removal of MM3s [21, 25, 38]. Although there are studies reporting that the angulation of impacted third molars is not a risk factor for inferior alveolar nerve damage [34, 39], there are also studies determining that angulation is associated with inferior alveolar nerve damage [30, 32]. Although Rood & Shehab suggested seven radiographic signs to predict the closeness between MM3 and IAN, four of these signs were found to be more effective in detecting the relationship [14]. Furthermore, Flygare and Ohman [40] reported that PR is generally acceptable for assessing the distance between MM3 roots and IAN in cases where there is no overlap between the two. Luo et al. [6] proposed that the close relationship between MM3 and IAN via the PR could be enough to anticipate future hypoesthesia of the chin or lip. Based on their study findings, they concluded that having a quality PR image and well-planned surgery can effectively reduce the likelihood of damage to the IAN.

While PR has traditionally been the main radiographic method used to assess different risk factors related to MM3 extraction, the development of additional methods has made it essential to compare these approaches with PR [20]. European guidelines advise the use of presurgical CBCT evaluation when the two-dimensional image indicates a significant proximity between the MM3 roots and the mandibular canal [41].

CBCT images provide valuable data for evaluating the relationship between MM3 roots and IAN in three dimensions. Additionally, these three-dimensional images are beneficial for preoperative planning and identifying alternate surgical methods [42]. Several previous studies have examined the characteristics of PR and CBCT in relation to impacted MM3s and IAN [1, 7, 20, 32, 43]. Multiple studies have consistently indicated that CBCT is superior to PR in assessing the relationship between the IAN and MM3 [7, 13, 35, 43]. Research has indicated that the additional information obtained from CBCT scans might potentially alter the surgical technique, leading to a decreased likelihood of damage to the IAN [26, 33, 35, 42]. Conversely, several studies have found that CBCT does not have any impact on either the treatment plan or the outcome of patients after surgery [1, 19, 34]. Surgical planning with CBCT for the removal of MM3s was found to have minimal impact on permanent IAN damage at long-term follow-up [33, 42]. Matzen et al. [19] reported that preoperative radiography techniques alone do not have an impact on the occurrence of IAN injuries. However, they noted that other factors, such as the anesthetic method employed during surgery, might potentially lead to IAN damage. The skill and proficiency of the performing surgeon have a significant impact on the extent of the IAN injury [43]. However, several oral and maxillofacial surgeons routinely obtain CBCT before MM3 surgery to eliminate legal liability [34].

The increasing complexity of cross-sectional imaging techniques plays a crucial role in the diagnosis of conditions related to third molars, which can be challenging for inexperienced radiologists in dental imaging [44]. Comprehensive knowledge of the imaging features of these abnormalities assists the practicing radiologist in achieving an accurate diagnosis, thus improving patient care [45]. CBCT enables detailed evaluation of impacted molars, odontogenic lesions, and jaw malformations. The angle of the impacted tooth and the distance between MM3 and IAN can be accurately determined in CBCT images without magnification [44–46]. CBCT imaging is often characterized by a longer time needed and a higher cost compared to two-dimensional radiographs. The mentioned cost depends not only on the high price of the CBCT device but also on the need to employ additional personnel [23, 47]. Petersen et al. [48] discovered that the cost of CBCT examinations is three to four times higher than the cost of PR examinations. Furthermore, it is crucial to acknowledge that while CBCT has a lower radiation dosage in comparison to spiral CT, it nevertheless subjects the patient to a higher level of radiation when compared to conventional radiographs [22, 23].

The utilization of CBCT is less common, particularly in developing countries, as a result of socioeconomic circumstances. While PR does not permit buccolingual dimensional assessment, it can nonetheless serve as the only radiographic examination technique prior to MM3 surgery in developing countries [15, 34]. Hence, it has been proposed that, if feasible, other methods with lower radiation levels should be taken into account to assess the radiationship between MM3 roots and IAN [33, 41].

When looking at the maxillofacial area, PA cephalometric radiographs are very important because they provide valuable mediolateral information, which is essential for evaluating the transverse relationships between craniofacial skeleton and dentoalveolar structures. Furthermore, PA cephalometric radiographs are characterized by their inexpensive cost and low radiation dose. PA cephalometric radiographs offer unique diagnostic insights that are not attainable by other two-dimensional imaging techniques [49]. Nevertheless, due to the nature of PA radiography being a two-dimensional technology, the picture is subject to distortion and projection. This impacts the precision of linear measurements acquired from PA radiographs. However, PA radiography may be utilized to compare the structures on both sides of the head, as they are positioned at about similar distances from the film and X-ray source [23]. PA radiographs can be used to determine the relationship between impacted MM3 and IAN [18]. In this study, the sensitivity and specificity of PA in predicting nerve exposure were 96% and 88%, respectively. The rates in question exhibit a significant disparity when compared to PR, the alternative approach assessed in this study.

PA radiography is not commonly performed as part of the standard diagnostic and treatment planning processes. Hence, there is a possibility of inaccuracies in the interpretation and recognition of anatomical landmarks on PA radiographs. Particularly in individuals aged 11 to 15, the superimposition of third molars might make it challenging to detect dental landmarks [50]. These are the limitations of using PA radiographs [51]. However, it has been reported that the occurrence of interpretation mistakes may be diminished with the use of precise definitions and comprehensive training [52]. According to Major et al. [53], operator expertise plays a crucial role in recognizing anatomical landmarks in radiological images due to their increasing familiarity with these appearances. Tai et al. [51] conducted research to assess the reliability of transverse dimensions in PA and CBCT. The study found no significant bias in intraobserver agreement. When the mandibular molar region was evaluated with PA, interobserver agreement was reported to be excellent [52]. Likewise, there was a significant level of agreement among observers in this study.

Obtaining both CBCT and PA radiography from a patient to assess their reliability raises ethical concerns. Consequently, the main limitation of this study is that it is a retrospective archival analysis with a relatively limited sample size. Therefore, it was a study in which only radiographic findings were evaluated, without clinical results, and since the study was an archive analysis, we did not have a role in improving the image quality by minimizing errors that may occur during radiographic imaging. In addition, in our study, only the presence of a relationship between MM3 roots and IAN was evaluated. A detailed classification based on radiographic signs (such as Rood and Shehap’s signs) and the correlation between patient age and MM3 and IAN were not evaluated. Future studies will contribute to the literature by investigating the radiographic signs that are used to determine the relationship between MM3 and IAN in PA radiographs.

## Conclusions

CBCT is unequivocally superior to other procedures due to its ability to assess the interrelationships of anatomical components in three dimensions without overlap and with little dimensional magnification. However, considering the accessibility in developing countries, the cost and radiation dose of CBCT, and due to their handicap, false positive relationship diagnosis of PR, PA radiography may be a reasonable option to evaluate the relationship between MM3 roots and IAN by providing mediolateral information.

## Data Availability

The datasets generated and/or analyzed during the current study are not publicly available due to confidentiality of personal information but are available from the corresponding author on reasonable request.

## Abbreviations

MM3: Mandibular third molar
IAN: Inferior alveolar nerve
PR: Panoramic radiograph
CBCT: Cone beam computed tomography
PA: Posteroanterior
